# Changes in subjective well-being and stress of older adults before, during and after the COVID-19 pandemic: a longitudinal study in Switzerland

**DOI:** 10.1007/s00127-024-02706-1

**Published:** 2024-07-06

**Authors:** Ryser Valérie-Anne, Gondek Dawid, Voorpostel Marieke

**Affiliations:** FORS C/O University of Lausanne, Bâtiment Géopolis, 1015 Lausanne, Switzerland

**Keywords:** Life satisfaction, Affect, Trajectories, Growth curve analysis, Swiss Household Panel

## Abstract

**Purpose:**

Despite the concerns about older adults’ overall quality of life during the COVID-19 pandemic, they often demonstrated better resilience, adaptability, and subjective well-being (SWB) than younger individuals. However, longer-term trends remain unclear. This study aims to describe older adults' trajectories in SWB dimensions before, during, and after the pandemic spanning 2017–2022.

**Methods:**

This study used piecewise growth curve analysis on a subsample of the Swiss Household Panel to investigate the population-average (n individuals = 3086; n = observations = 13,780) trajectories of SWB dimensions and stress among adults aged 65 and older between 2017 and 2022. We also tested whether these trajectories differed by age, gender, and household income.

**Results:**

Life satisfaction and positive affect remained stable among older people during the pandemic (2019–2021) but declined after. Negative affect increased during the pandemic and decreased afterward, while stress levels increased slightly before and significantly after the pandemic. The trajectories did not differ by gender or household income, but the oldest-old (> 75-year-old) had a more significant decline in positive affect and life satisfaction pre-pandemic (2017–2019) and stress during the pandemic (2020–2021).

**Conclusion:**

Research shows that older adults possess adaptation skills and emotional competencies, which enable them to navigate pandemic challenges. However, we show that the post-pandemic era presents more substantial challenges for this older population, who perhaps face more difficulties adapting to the new uncertain post-pandemic world. Further research needs to examine if these findings replicate in other contexts, for instance, where pandemic containment measures have been more stringent.

## Introduction

The COVID-19 pandemic, a major 21st-century event, globally impacted everyone [1], particularly older adults who faced higher risks from the virus [2]. Despite their vulnerability and the need for strict social distancing, studies have shown that older adults have impressively adapted to the disruptions caused by the pandemic [3, 4].

Studies tend to show relative stability in life satisfaction [5, 6] and mental health [7], with no significant divergence from the trends observed before the pandemic. However, there is also some evidence of increased negative affect among older people [8]. This is in contrast to younger people who tended to report an increase in stress [9], depressive and anxiety symptoms [10], and declines in life satisfaction [11] and negative affect [12]. This suggests better adaptation among older people compared with younger age groups in the early stages of the pandemic [13]. Nonetheless, the evidence on longitudinal trajectories starting pre-pandemic is limited [14, 15]. Hence, it is difficult to determine whether the changes observed during the pandemic continue a longer trend [14, 15]. There is also inconsistency in evidence on subjective well-being (SWB) trajectories later during the pandemic, with some studies finding a deterioration in different dimensions of SWB in the later stages of the pandemic [16] and worsened depression and anxiety [6, 15]. Other studies found stability in stress, negative affect, and depressive symptoms with positive affect [17]. Post-pandemic evidence seems virtually lacking.

Our study aims to describe population-average trajectories of SWB and stress of older adults living independently between 2017 and 2022. To further explore potential heterogeneity in the trajectories, we compare the ‘young-old’ (65 to 74 years) and the ‘oldest-old’ (75 years and above).

## Method

### Data, sample

This study draws on longitudinal data from six waves (2017–2022) of the Swiss Household Panel-SHP [18, 19]. This nationally representative survey interviewed all household members older than 14 years of age from a random sample of private households in Switzerland since 1999. Questionnaires were administered mainly by computer-assisted telephone interviewing (CATI) (96–98%), with the rest of the participants using computer-assisted web interviewing (CAWI).

The analytical sample includes a subsample of SHP participants who were at least 65 years old in 2019 and had at least one valid measure of SWB between 2017 and 2022 (n individuals = 3086; n observations = 13,780). Table 1 provides participants’ descriptive information.

Among the entire sample, 49.6% had no missing information across the dependent variables, meaning they had completed information across all six waves. Additionally, 12.6% had only one measure of dependent variables across the time points (out of six possible). The missing data pattern was monotone, with the number of participants having missing outcomes increasing by 6.0–7.5% each observation year (2017–2022) (see Table 2).

### Measures

#### Dependant variables

Two components of SWB—positive affect and life satisfaction (PALS) and negative affect (NA)—were derived by summing up the relevant items ranging from 0 to 10. A higher score indicated greater satisfaction in each life domain and more frequent positive and negative affect. Table 3 details these measures. The psychometric properties of both measures were extensively examined [20], showing high internal consistency and scalar measurement invariance across time, age, sexes, and survey modes.

Positive affect and life satisfaction (PALS) comprised six items: (1) life satisfaction, (2) satisfaction with health, (3) satisfaction with personal, social, and family relationships, (4) satisfaction with leisure time activities [21, 22]; and two positive affect items [23] (5) the level of energy and optimism and (6) the level of joy. The internal consistency was strong, ranging between 0.75 and 0.76 across study years. The PALS score could range from 0 to 60.

Negative affect (NA) [23] comprised four items that assessed the level of four negative emotions—anger, sadness, worry/anxiety, and depression. The internal consistency was strong, ranging between 0.75 and 0.77 across study years. The NA could range from 0 to 40.

Stress [24] was measured using a single item that assessed an individual’s stress level, ranging from 0–“never” to 5–“very often.”

#### Covariates

Sociodemographic indicators were included as potential effect modifiers of the age-SWB association to examine differences in the population-average trajectories across subgroups of the population: gender (1 = male; 2 = female), age groups (aged 65–74 years; those aged 75 years and above), and equivalized household income in quartiles.

### Statistical analysis

The longitudinal population-average trajectories in SWB in 2017–2022 were described using piecewise growth curve analysis. This approach accounts for the multilevel structure of longitudinal data, where occasion-specific measurements are nested within individuals, and incomplete or unbalanced data, taking advantage of maximum likelihood estimation [25]. Our model included both fixed effects and random effects. Fixed effects represent the population’s average effect of time while controlling for age (and age^2^). Random effects include information about variance around the starting point of the trend (an intercept) and the trend itself (a slope).

Time is conceptualized as four separate periods (slopes), representing an overall trend between 2017 and 2022. The four slopes were (1) pre-pandemic—2017–2019; (2) into-pandemic—2019–2020; (3) pandemic—2020–2021; and (4) out-of-pandemic—2021–2022. This allowed us to directly compare the change in different periods. For instance, we could test whether a potential decline in SWB before the pandemic equaled a potential improvement in SWB post-pandemic using a Wald test, which formulates the null hypothesis that these two slopes equal zero (i.e., slope_2017–2019_ − slope_2021–2022_ = 0).

We also compared the trajectories of SWB between men and women, age groups (65–75 vs > 75-year-old), and quartiles of the equivalised household income by allowing the slopes to vary by these groups. We did this by including interaction terms between the age groups and slopes (e.g., gender*slope_2017–2019_, gender*slope_2019–2020,_ gender*slope_2020-2021_, gender*slope_2021–2022_), and testing for differences with the Wald test (at p < 0.05). We further investigated the differences by running pairwise comparisons of estimated marginal means. All models were also controlled for the survey mode (CATI, CAWI).

## Results

### Positive affect and life satisfaction (PALS)

**Fig. 1:**
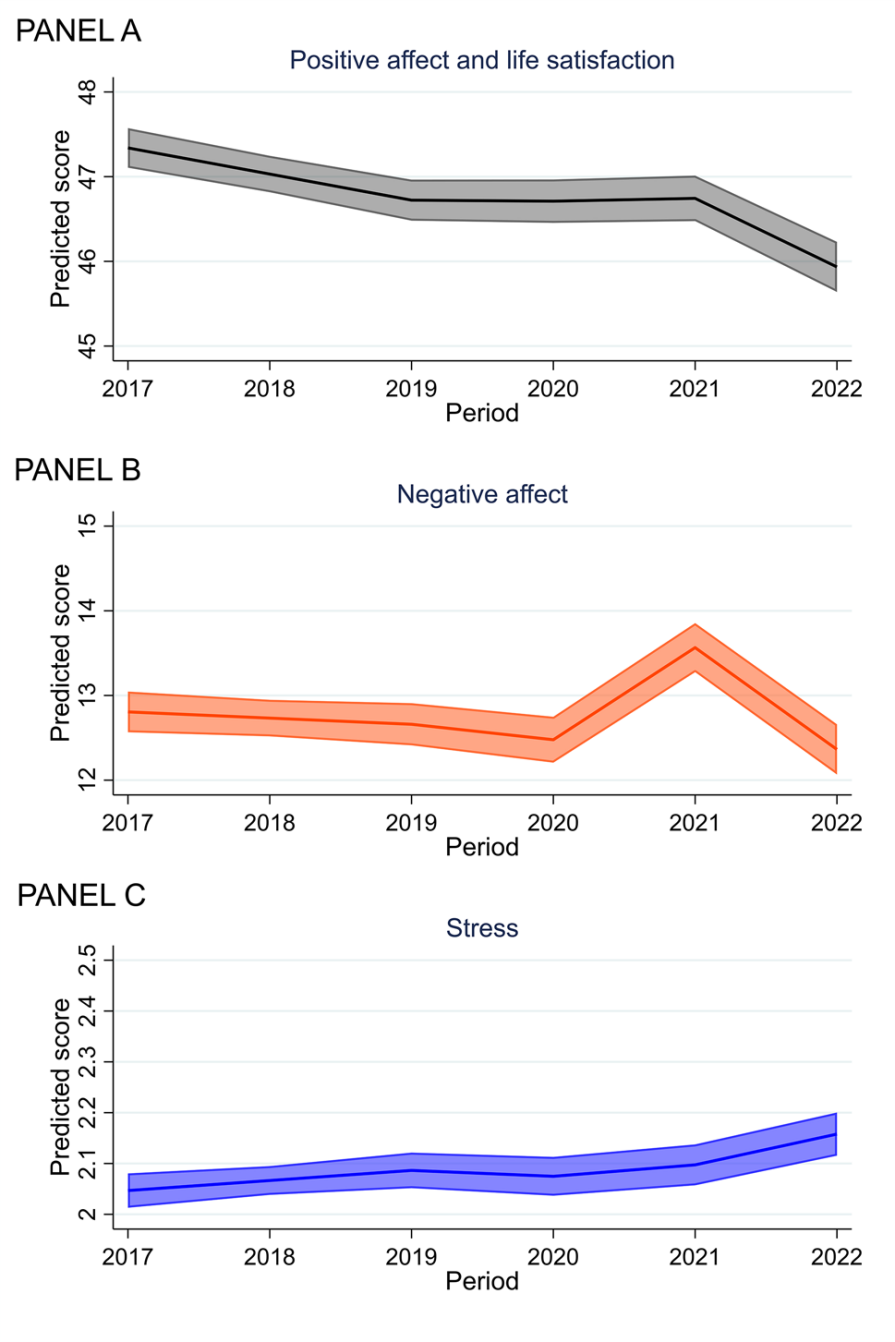
Average trajectories of frequency of positive affect and life satisfaction (Panel A), negative affect (Panel B), and stress (Panel C). The observed score of PALS ranged 5–60 (possible range was 0–60). The possible and observed score of NA ranged 0–40

PALS somewhat decreased before the pandemic (−0.31, −0.41 to −0.21), was stable into (−0.01, −0.20 to 0.17) and during the pandemic (0.03, −0.17 to 0.24), then it declined out-of-pandemic (−0.81, −1.04 to −0.58) (see Fig. 1 Panel A; based on model estimates from Table 4). The oldest respondents expressed a greater decline in PALS between 2017 and 2019 (−0.57, −0.74 to −0.40) than the young (−0.20, −0.32 to −0.09). We found no evidence for differential trajectories across gender and income.

**Fig. 2:**
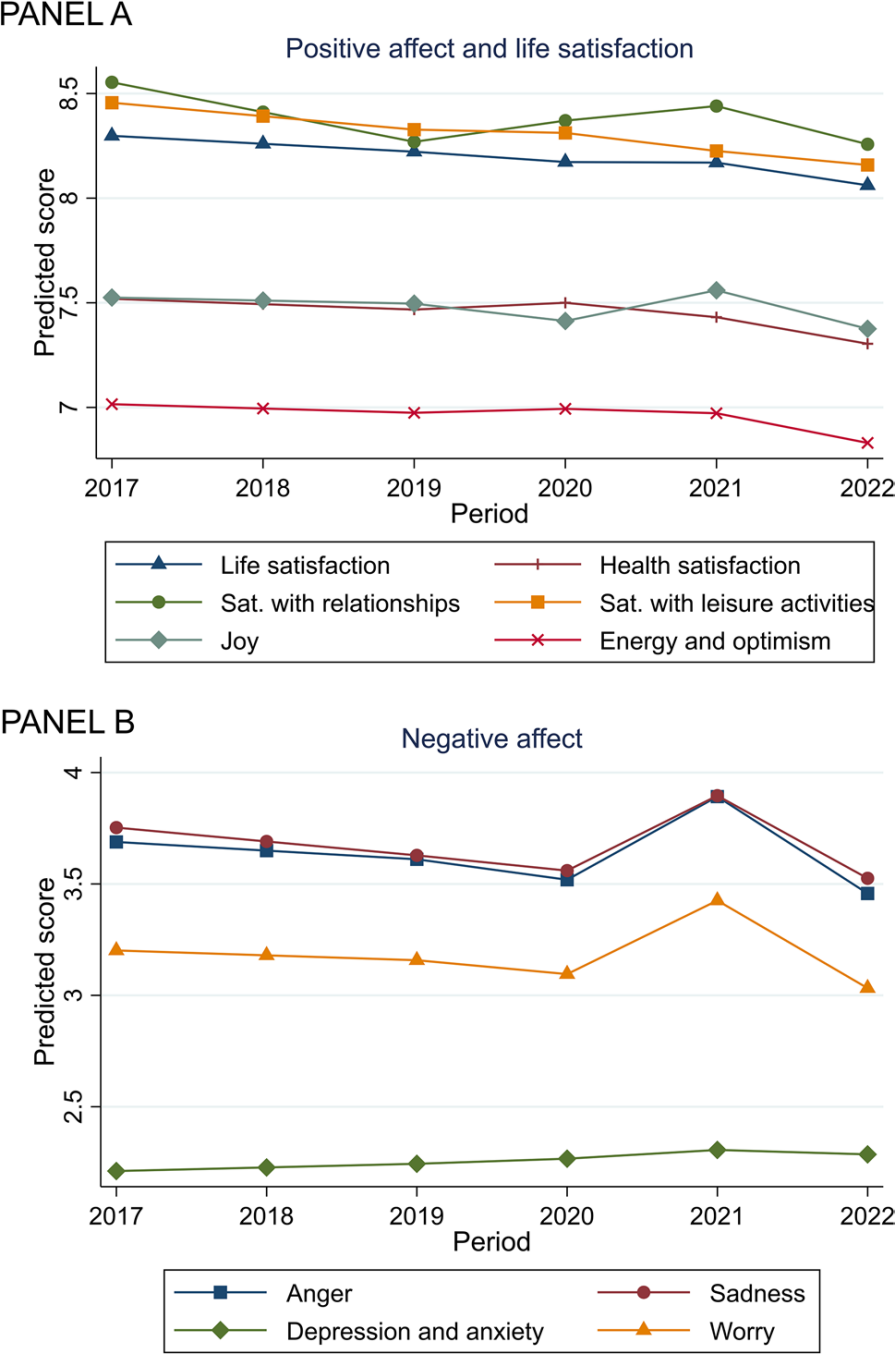
Average levels of single items spanning from 2017 and 2022

When examining the items separately (Table 4; Fig. 2 Panel A), satisfaction levels with relationships and joy improved during the pandemic. At the same time, they were stable for life satisfaction, energy, and optimism, but all declined after the pandemic. The drop in relationship satisfaction was greater out-of-pandemic (−0.18, −0.25 to −0.12) than the increase reported during the pandemic (0.07, 0.01–0.13). Satisfaction with health decreased, on average, both during (−0.07, −0.14 to 0.00) and out-of-pandemic (−0.13, −0.20 to −0.05).

### Negative affect (NA)

The average levels of NA were stable before (−0.07, −0.19 to 0.04) and into-pandemic (−0.18, −0.40 to 0.04) but increased drastically during the pandemic (1.09, 0.84–1.33) and declined out-of-pandemic (1.09, 0.84–1.33) (Fig. 1 Panel B; based on model estimates from Table 4). We found no evidence for differential trajectories across gender, age, and income.

The average frequency of anger, sadness, and worry increased during the pandemic and declined equivalently out-of-pandemic. For instance, for worry, the increase in 2020–2021 was 0.33 (0.23–0.43), and the subsequent decline (2021–2022) was −0.39 (−0.50 to −0.29) (Fig. 2 Panel B).

### Stress

The average stress levels marginally increased two years before the pandemic (0.02, 0.00–0.04). Subsequently, they were relatively stable into- (−0.10, −0.05 to 0.03) and during the pandemic (0.02, −0.02 to 0.07) and increased again out-of-pandemic (0.06, 0.02–0.11) (Fig. 1 Panel C; based on model estimates from Table 4). The oldest-old respondents had a greater increase in stress between 2020 and 2021 (0.09, 0.01–0.16) than young-old (−0.02, −0.07 to 0.03). We found no evidence for differential trajectories across gender and income.

## Discussion

The post-pandemic decline in PALS and increase in stress are multifaceted. While older adults possess relatively good competencies for adjusting to short-term events, they struggle with long-term stressors [13]. Thus, the prolonged nature of the pandemic’s impact may have contributed to the worsening of SWB among older individuals. Additionally, the rise in societal ageism [26] during the pandemic might have contributed to this decline in PALS and increase in stress, as negative stereotypes and perceptions about older people have intensified, harming their quality of life [27, 28]. Moreover, despite the heightened focus on older adults as a vulnerable group during the pandemic, they faced significant challenges (loss of close relationships, enforced isolation, and reduced social interactions), which exacerbated feelings of loneliness and isolation. These experiences have negatively affected their mental health [28] and may have enduring impacts on their PALS.

The escalating negative emotions during the 2020–2021 pandemic align with some previous findings of increased depression and anxiety [15] but not with others, suggesting that older adults experienced less negative affect [12, 29]. However, we observed a post-pandemic decline in negative emotions to pre-pandemic levels. This pattern, evident in specific feelings like anger, sadness, and worry, may stem from pandemic-related stressors such as health concerns, isolation, and frustration over restrictions. With the pandemic’s decrease in threat, these negative emotions have reverted to baseline, indicating a direct emotional response to the pandemic’s challenges and subsequent adaptation.

Most older adults were resilient early in the COVID-19 pandemic [e.g. 3, 4, 13, 14, 28]. However, our study revealed that they faced increasing difficulties over time, affecting their SWB and stress levels. While changes were minor but statistically significant, their broad impact is notable due to the many older adults affected.

Despite many strengths, our study presents some limitations. The most notable one is the potential bias resulting from missing information. As in most observational studies [30], there was a dropout, likely among those with poor physical and/or cognitive health and limited economic resources. We were also unable to explore the sensitivity of our findings to survival bias. Moreover, our study did not include participants in assisted living or care facilities who had not had the same experience of the pandemic as their counterparts living at home. In the Swiss context, many individuals in medical and social care establishments were isolated during the pandemic due to the prohibition of family or friend visits, as in hospitals. Thus, both isolation and lack of social relationships negatively affect SWB trajectories [28]. Their experience was very different from that of people living at home. During the pandemic, Switzerland introduced, compared with other countries, a semi-lockdown with only open shops, but non-essential shops and public places were closed. However, outdoor activities were permitted, provided that no more than five people were present and that physical distance was maintained. The whole population, especially the older adults and those at risk were encouraged to stay at home. However, thanks to private initiatives, it was possible for vulnerable people to ask for help, particularly for shopping, if they did not want to take the risk of going out. The possibility of asking for help and seeing up to five other people, even if it was outside, encouraged some older people to continue social activities.

Furthermore, the measure of socioeconomic inequality in our study, the equivalized household income, did not adequately consider accumulated wealth. It is possible that some individuals with extensive wealth were classified as having low income, making the comparison groups highly heterogeneous. This could have led to diluting any differences in trajectories across income quartiles.

To conclude, our findings emphasize that despite largely resilient adaptation to the pandemic’s stressors, the post-pandemic period presented, somewhat unexpectedly, significant challenges for older adults regardless of gender, age group, or financial resources.

## Appendix

See Tables 1, 2, 3, 4 and Fig. 2.

**Table 1:** Demographic characteristics of the SHP participants (65-year-old or older) (n = 3086)

	N	Missing n (%)	Proportion n (%)
Gender	3086	0 (0.0)	0.0
Men			1414 (45.8)
Women			1672 (54.2)
Age groups	3086	0 (0.0)	
Young-old (age 65–74)			1843 (59.7)
Oldest-old (age > 75)			1243 (40.3)
Income (quartile)	2284	802 (25.5)	
1^st^			771 (33.8)
2^nd^			545 (23.9)
3^rd^			500 (21.9)
4^th^			468 (20.5)

	N	Missing n (%)	Mean (SD; range)
Age	3086	0 (0.0)	74.70 (6.93; 65–99)
PALS	13,650^a^	4866 (26.3)	47.01 (6.47; 5–60)^b^
Negative affect	13,699^a^	4817 (26.0)	12.67 (6.81; 0–40)
Stress	13,761^a^	4755 (25.7)	2.09 (0.95; 1–5)

Sources: Swiss Household Panel (wave 2017–2022); authors’ calculations

*PALS* positive affect and life satisfaction

^a^This refers to the number of observations

^b^This refers to the observed range. Possible range was 0–60

**Table 2:** The proportion of individuals with missing outcome at each time point

N total = 3086^a^	PALS	Negative affect	Stress
	n (%) missing	n (%) missing	n (%) missing
2017	2790 (90.4)	2789 (90.4)	2803 (90.8)
2018	2605 (84.4)	2607 (84.5)	2619 (84.9)
2019	2373 (76.9)	2377 (77.0)	2400 (77.8)
2020	2183 (70.7)	2195 (71.1)	2198 (71.2)
2021	1950 (63.2)	1964 (63.6)	1969 (63.8)
2022	1749 (56.7)	1767 (57.3)	1772 (57.4)

Sources: Swiss Household Panel (wave 2017–2022); authors’ calculations

^a^This refers to the number of individuals

**Table 3:** Details about the measures of subjective well-being

Subjective well-being	Questions	Response options	Source
Life satisfaction domains and positive affect			
	Life satisfaction: in general, how satisfied are you with your life?	0 (not at all satisfied)–10 (completely satisfied)	Source: adapted from http://internal.psychology.illinois.edu/~ediener/SWLS.html Reference: Diener, Ed, Robert A. Emmons, Randy J. Larsen, and Sharon Griffin. 1985. “The satisfaction with life scale.” Journal of Personality Assessment 49(1):71–75 [21]
	Health satisfaction: how satisfied are you with your state of health, if 0 means "not at all satisfied" and 10 "completely satisfied"?	0 (not at all satisfied)–10 (completely satisfied)	Based on the conceptualization of Diener, Ed, Mark Eunkook Suh, Richard E. Lucas, and Heidi L. Smith. 1999. “Subjective well-being: three decades of progress.” Psychological Bulletin 125(2):276–302
	Relationships satisfaction: how satisfied are you with your personal, social and family relationships?	0 (not at all satisfied)–10 (completely satisfied)	Designed for the purpose of the Swiss Household Panel
	Leisure time satisfaction: how satisfied are you with your leisure time activities?	0 (not at all satisfied)–10 (completely satisfied)	
	Energy and optimism: are you often plenty of strength, energy and optimism?	0 (never)–10 (always)	Based on the conceptualization of Watson, David, Lee A. Clark, and Auke Tellegen. 1988. “Development and validation of brief measures of positive and negative affect: The PANAS scales.” Journal of Personality and Social Psychology 54(6):1063–70
	Joy: how frequently do you generally experience the following emotions?	0 (never)–10 (always)	Scherer, Klaus R., Wranik, Tanja, Sangsue, Janique, Tran, Veronique, and Scherer, Ursula. 2004. “Emotions in everyday life: probability of occurrence, risk factors, appraisal and reaction patterns.” Social Science Information, 43, 499–570 [23]
Negative affect			
	Anger: how frequently do you generally experience the following emotions?	0 (never)–10 (always)	Scherer, Klaus R., Wranik, Tanja, Sangsue, Janique, Tran, Veronique, and Scherer, Ursula. 2004. “Emotions in everyday life: probability of occurrence, risk factors, appraisal and reaction patterns.” Social Science Information, 43, 499–570 [23]
	Sadness: how frequently do you generally experience the following emotions?	0 (never)–10 (always)	
	Worry: how frequently do you generally experience the following emotions?	0 (never) – 10 (always)	
	Anxiety and depression: do you often have negative feelings such as having the blues, being desperate, suffering from anxiety or depression?	0 (never)–10 (always)	Based on the conceptualization of Watson, David, Lee A. Clark, and Auke Tellegen. 1988. “Development and validation of brief measures of positive and negative affect: The PANAS scales.” Journal of and Social Psychology 54(6):1063–70
Stress measure			
	Stress: how often have you felt stressed during the last month?	1 (Never) 2 (Almost never) 3 (Sometimes) 4 (Fairly often) 5 (Very often)	Item adapted from Cohen, S., Kamarck, T., & Mermelstein, R. (1983). A global measure of perceived stress. Journal of Health and Social Behavior, 24, 386–396 [24]

**Table 4:** The population average trajectories across study outcomes—model estimates

	PALS	Negative affect	Stress
	Coefficient (95% CI)	Coefficient (95% CI)	Coefficient (95% CI)
Period 2017–19	−0.31 (−0.41, −0.21)	−0.08 (−0.19, 0.04)	0.02 (0.00, 0.04)
Period 2019–20	−0.01 (−0.19, 0.18)	−0.18 (−0.40, 0.04)	−0.01 (−0.05, 0.03)
Period 2020–21	0.04 (−0.16, 0.24)	1.10 (0.86, 1.35)	0.02 (−0.02, 0.06)
Period 2021–22	−0.80 (−1.03, −0.57)	−1.21 (−1.46, −0.96)	0.06 (0.01, 0.10)
Age	−0.04 (−0.07, −0.01)	0.03 (−0.00, 0.06)	−0.01 (−0.01, −0.00)
Mode of interview			
CATI (reference category)	–	–	–
CAWI	−0.64 (−1.25, −0.02)	−0.45 (−1.15, 0.26)	0.15 (0.05, 0.24)
Random intercept—variance	30.57 (28.43, 32.88)	29.03 (27.38, 30.77)	0.43 (0.40, 0.46)

Sources: Swiss Household Panel (wave 2017–2022); authors’ calculations

*CI* confidence intervals, *CATI* computer-assisted telephone interviewing, *CAWI* computer-assisted web interviewing

## Data Availability

The “Living in Switzerland Waves 1-24 (including a long file) + Covid 19” data supporting this research's findings are available from SwissUbase to the scientific community upon submission of a data request application (https://www.swissubase.ch). Study data has already been de-identified.
